# Supplementary material for: Prostaglandin F2α Regulates Adipogenesis by Modulating Extracellular Signal-Regulated Kinase Signaling in Graves’ Ophthalmopathy
Source: Int J Mol Sci. 2023 Apr 10;24(8):7012. doi: 10.3390/ijms24087012 (PMC10138945; doi:10.3390/ijms24087012)
Supplement: Supplementary file 1 [file ijms-24-07012-s001.zip › ijms-2280401-supplementary.pdf]

Supplementary Information

Prostaglandin F2α Regulates Adipogenesis by Modulating Extracellular Signal-Regulated Kinase Signaling in Graves' Ophthalmopathy

Materials and Methods

1. Materials

Dulbecco's modified Eagle medium (DMEM), 0.25% trypsin-ethylene diamine tetraacetic acid (EDTA) (1x), and penicillin-streptomycin mixture were purchased from ThermoFisher Scientific (Carlsbad, CA, USA). Fetal bovine serum (FBS) was purchased from Procell (Wuhan, China). Biotin, pantothenic acid, rosiglitazone, transferrin, triiodothyronine (T3), dexamethasone, insulin, and 3-Isobutyl-1-methylxanthine (IBMX) were purchased from Sigma-Aldrich (Saint Louis, MI, USA).

PGF2α, Ebopiprant (an FPR antagonist), and U0126 (an ERK inhibitor) were purchased from MedChemExpress (Monmouth Junction, NJ, USA).

2. Primary Culture and Adipogenic Induction of OFs

Orbital adipose tissue samples were collected and washed three times with phosphate-buffered saline (PBS). They were cut into small pieces of 1–2 mm^3 and distributed evenly on the bottom of a culture flask. An appropriate amount of DMEM medium was added, and the tissue was cultured in a cell incubator at 37°C and 5% CO_2. When 80% of the bottom of the flask was occupied, the cells were digested with 0.25% trypsin for passage. Cells from passages 4 to 8 were used for subsequent experiments. Immunofluorescence staining of the OFs was performed as previously described[1]. The primary antibodies used were vimentin (1:500; Abclonal, Wuhan, China), cytokeratin (1:500; Abclonal, Wuhan, China), desmin (1:500; Abclonal, Wuhan, China), S-100 (1:500; Abclonal, Wuhan, China), and myosin (1:500; Abclonal, Wuhan, China). The secondary antibody was FITC-conjugated Goat Anti-Rabbit IgG (H+L) (1:200; Servicebio, Wuhan, China).

3. Reverse Transcription-Polymerase Chain Reaction (RT-PCR)

Total RNA was extracted from OF cells using the RNA quick purification kit (Omega Biotek, Norcross, GA, USA) according to the manufacturer's instructions, and RNA was reverse transcribed to complementary DNA (cDNA) using the PrimeScript RT kit (Vazyme, Nanjing, China). RT-PCR was performed using the SYBR Fast qPCR kit (Vazyme, Nanjing, China). The housekeeping gene, glyceraldehyde phosphate

dehydrogenase (GAPDH), was used as an internal control. Supplementary Table S1 lists the primer sequences.

Figure

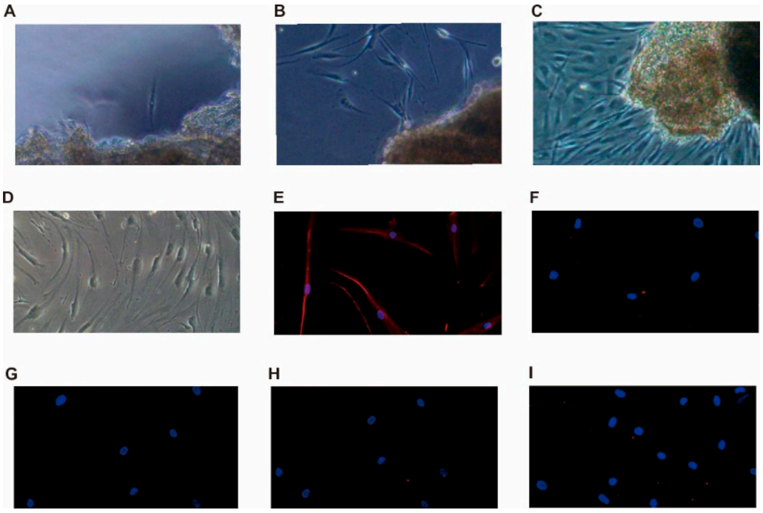

Figure S1. Primary Culture and Identification.

Adherent ocular tissues:(A)3 day; (B)5 day; (C)10 day; (D) OFs cell morphology; (E) Vimentin (+); (F) Cytokeratin (-); (G) Desmin (-); (H) S-100 (-); (I) Myosin (-); Pictures are shown at magnification ×100.

Table

Table S1. The Primer Sequences Used in RT-PCR.

**Gene**	**Forward 5' to 3'**	**Reverse 3' to 5'**
FPR	AAAAGTCAGCAGCACAGACAAGG	CAGAAATGGGCTCCAACAAATAC
PPARγ	AGCTGAAGCTGAACCACCCT	CGTGACAATCTGTCTGAGGTCTG
FABP4	AGAAGTAGGAGTGGGCTTTGC	GTCATCTGCAGTGACTTCGTC
GAPDH	TTAGCACCCCTGGCCAAGG	CTTACTCCTTGGAGGCCATG

Reference:

1. Feng JP, Zhu R, Jiang F, et al. Melittin-encapsulating peptide hydrogels for enhanced delivery of impermeable anticancer peptides. *Biomaterials science* 2020;8:4559-4569.
